# A Deep Dive into the Nexus between Digital Health and Life Sciences Amidst the COVID-19 Pandemic: An Editorial Expedition

**DOI:** 10.3390/life13051154

**Published:** 2023-05-10

**Authors:** Daniele Giansanti

**Affiliations:** Centre Tisp, Istituto Superiore di Sanità, 00161 Rome, Italy; daniele.giansanti@iss.it; Tel.:+39-06-49902701

I am proposing this editorial to briefly trace the evidences that emerged from the *Special Issue* (SI)—*The Digital Health in the Pandemic Era*—[1] that I had the pleasure of following in Life.

The idea of developing this Special Issue was born when the COVID-19 pandemic was still having a major impact on the health of the planet [2].

As is well known, *digital health* (DH) encompasses a diverse range of technologies, including wearable and internal devices, various types of sensors, and innovative solutions. DH can facilitate the identification of health risks and provide assistance in the diagnosis, treatment, and monitoring of various health conditions. Generally, DH presents immense potential for both the general population and healthcare professionals.

Since the beginning of the pandemic [2], this technology has been applied to the *health domain*, and it has played a crucial role in providing remote assistance and continuity of care at home, thereby protecting patients, healthcare workers, limiting the spread of the virus, and reducing the need for hospitalization.

For instance, digital measurement of oxygen saturation at home has provided key decision-making data for patients’ health, such as choosing between hospitalization and respiratory support.

Additionally, remote monitoring of frail patients with underlying conditions, such as diabetes, cardiovascular disease, or oncological problems has improved the continuity of care and reduced pressure on hospitals. Digital contributions have affected the fight against the pandemic in numerous ways, such as managing digital contact tracing and vaccination processes using smart technology.

The SI:

- Explored innovations in the field of DH stimulated by the COVID-19 pandemic, and the acceptance of this revisited DH by all, including stakeholders, healthcare professionals, and citizens.
- Also, analyzed the successes and failures of DH applications during the pandemic, highlighting the critical role played by remote health monitoring systems, the contribute of the Artificial Intelligence, and the potentials they could offer for post-pandemic healthcare delivery.

At the time of writing this editorial contribution, 18 papers have been published [1], including one editorial, one opinion, one review, one systematic review, one comment and one reply, and twelve scientific articles.

A quick overview of the contents of the published works demonstrates how the topic of DH, as faced and revisited during the pandemic, has played a connecting role for many of the branches of the life sciences [3], shown in Table 1.

Five scientific papers were published, including the editorial [2,4,5,6,7], equal to 27.8% of all the papers contained in the SI, which dealt with the topic of DH applied to contact tracing, the so-called digital contact tracing (DCT). These studies reported [4,5] and/or discussed [6,7] the impact of the DCT on the spread of the pandemic in different populations, together with the factors that influenced its use. The DCT, a new application of the DH, uses apps and various methods of tracking position and proximity, and it had a wide diffusion during the pandemic [2]. It has been consolidated as a technological method with great potential, applying digital solutions for automatic contact tracing, for both a punctual and a global tracking of the evolution of an epidemic. A strategic epidemiology activity was developed for tracking communicable diseases, as in the case of the COVID-19 pandemic. The DCT highlights an important role of DH in epidemiology as a major component of public health research in the branch of the life sciences (Table 1), studying factors affecting the health of populations.

All the other studies [8,9,10,11,12,13,14,15,16,17,18,19,20] have dealt with the use of DH in the life sciences, which, in addition to bioengineering, touched by all, have examined, from time to time, other categories (Table 1), such as Anatomy [8,11], Bioinformatics [14,16], Cell biology [8,11], Neuroscience [8,12,15,17,20], Physiology [9,10,12,13], Population biology [14,16,18,19], and others that are shown in Table 1.

The SI, in particular, highlights how the contribution of DH to the life sciences during the pandemic also took place with the support of artificial intelligence (AI), both in applications related to COVID-19 specific diagnostics, diagnostics in general, and on population surveys regarding biomedical aspects during the pandemic.

In [8], an application of AI in brain tumors integrated in diagnostic imaging is reported. Two studies, based on AI, have addressed aspects connected with the fitness and wellness of the population.

The first study applied AI, integrated with wearable devices, to monitor physical activity (which plays an important role in controlling obesity and maintaining healthy living) during the pandemic [9].

The second study applied IoT and AI [10] in investigating the risk factors and the ratio of obesity, proposing an approach for obesity diagnosis in its initial stages, significantly increasing the patient’s chances of effective treatment.

An AI algorithm for the early detection of abnormalities in chest X-rays, for COVID-19 diagnostics, was proposed in [11]. It used a deep hybrid learning-based framework for the detection of COVID-19 using chest X-ray images.

The importance of DH application in the physiological issues related to physical activity was investigated in [12,13], through population surveys. In [12], the authors tested the feasibility of virtually delivering an *exergame-based* physical activity intervention to older breast cancer survivors, while the authors of the study reported, in [13], that they investigated the changes experienced by Austrian therapists when switching to psychotherapy at a distance.

Large-scale population surveys have been conducted, both to analyze the impact of biomedical parameters and health determinants on the population, as well as digital literacy, key factors in using DH [14,15,16].

The study in [14] addressed the importance of *Big-medical-data classification and image detection* as crucial tasks in the field of healthcare. They proposed a specific algorithm for medical data classification and image detection in the COVID-19 era that may have significant implications in the *health domain*.

The outcome from “Understanding COVID” (a public health campaign designed in 2020 and launched in 2021 in Asturias in Spain to provide reliable and comprehensive information oriented towards vulnerable populations, which is also related to digital literacy) was reported in [15].

The study reported in [16], using AI, and particular feature selection approaches, evaluated the aspects affecting the health of students throughout the COVID-19 lockdown time period.

The importance and the impact of *mental health* monitoring through self-monitoring apps was addressed in a study reported in [17].

Two specific reviews addressed and overviewed the impact of DH in remote health care and healthcare interventions [18,19]. The impact of *eHealth* interventions on the improvement of self-care in chronic patients was overviewed in [18], while the healthcare professionals’ experience of reforming digital care visits was investigated in [19].

Finally, an opinion piece investigated the impact of chatbots in the health domain [20], reporting an increasing of their use during the pandemic, also thanks to the AI.

*In conclusion:*

The published works on the DH, reported in the collection [1,2,4,5,6,7,8,9,10,11,12,13,14,15,16,17,18,19,20], demonstrate the usefulness of DH in connecting various branches of life sciences, especially during the pandemic. The use of DH in contact tracing has been particularly significant, with several studies reporting on its impact and factors influencing its use [2,4,5,6,7]. In fact, the application of DH in digital contact tracing (DCT) has emerged as a major component of public health research, showcasing its potential role in epidemiology, a major component of the life sciences (Table 1).

Additionally, DH applications have been used in biomedical diagnostics [8,11], physical activity [12,13], mental health monitoring [17], and remote healthcare interventions [12,13,14,15,16,17,18,19].

Large-scale population surveys have also been conducted to analyze the impact of biomedical parameters, health determinants, and healthcare literacy on the population [14,15,16], while chatbots have increasingly been used in the health domain [20].

Moreover, the support of Artificial Intelligence (AI) has further amplified the impact of DH in diagnostics [8,11], physical activity monitoring [12], obesity diagnosis [12], and healthcare interventions and determinants on the population [12,13,14,15,16,17,18,19].

DH has effectively aided public health research related to studying factors affecting the health of populations, while also contributing to advances in various fields of the life sciences (Table 1), such as, Biongineering [8,9,10,11,12,13,14,15,16,17,18,19,20], Anatomy [8,11], Bioinformatics [14,16], Cell biology [8,11], Neuroscience [8,12,15,17,20], Physiology [9,10,12,13], and Population biology [14,16,18].

Overall, this highlights that:

- DH has played and plays the role of a connector among different branches of life sciences, particularly in times of crisis.
- The high-quality research in this area remarks the usefulness of DH as a powerful tool for scientific and medical research.

## Figures and Tables

**Table 1:** Branches of the life sciences.

Branches of Life Sciences
Anatomy, Biochemistry, Bioengineering, Bioinformatics, Biophysics, Biotechnology, Botany, Cell biology, Developmental biology, Ecology, Entomology, Epidemiology, Ethology, Evolutionary biology, Genetics, Hematology (also known as Haematology), Microbiology, Molecular biology, Neuroscience, Physiology, Population biology, Structural biology, Toxicology, Zoology

## Data Availability

Not applicable.
